# Role of Oxytocin in the Pathogenesis and Modulation of Borderline Personality Disorder: A Review

**DOI:** 10.7759/cureus.13190

**Published:** 2021-02-07

**Authors:** Muhammad Youshay Jawad, Bakhtawar Ahmad, Ali Madeeh Hashmi

**Affiliations:** 1 Academic Department of Psychiatry and Behavioral Sciences, King Edward Medical University/Mayo Hospital, Lahore, PAK

**Keywords:** oxytocin, borderline personality disorder, clinical neuroscience, differential susceptibility

## Abstract

Borderline personality disorder (BPD) is a serious psychiatric condition characterized by dysfunctional relations, abnormal social behavior, and high morbidity. Many studies have implicated abnormal oxytocinergic system as a causative factor of behavioral dysregulation in BPD patients. The objective of this review is to provide a comprehensive analysis of the association of oxytocin with the pathogenesis of BPD and its possible role as a therapeutic agent. Our review indicates that a combination of genetic and environmental factors causes BPD patients to have lower baseline levels of oxytocin, leading to increased activation of the amygdala. This results in defective cognition of social stimuli, leading to abnormal behaviors like affective instability, unresolved attachment, and emotional dysregulation. Clinical trials conducted on BPD patients using intranasal oxytocin have shown both prosocial and trust-lowering effects. The effects of oxytocin depend upon various patient characteristics like the history of childhood trauma and the nature of attachment. Even though evidence of oxytocin's role in modulating behavior in BPD patients already exists, further studies are required to more clearly elaborate on this role to fully explore oxytocin's potential as a therapeutic agent.

## Introduction and background

People suffering from personality disorders form the major share of patients receiving psychiatric care, and among all personality disorders, borderline personality disorder (BPD) is the most commonly treated issue along with schizotypal personality disorder [1]. The psychopathological domains of BPD can be very well illustrated along three lines: disturbed relatedness (unstable relationships, identity disturbance, and a chronic feeling of emptiness); behavioral dysregulation (impulsivity and suicidal/self-mutilative behavior); and affective dysregulation (affective instability, inappropriate anger, and efforts to avoid abandonment) [2]. Affective instability refers to multiple mood changes within a single day, ranging from euphoria to dysphoria, and anger outbursts followed by feelings of guilt often targeted toward caregivers [3]. This affective instability was the most common criterion present at baseline as well as after two years in clinically diagnosed BPD patients as per a study [4]. BPD patients face stigma not only in society but in psychiatric and mental health units as well. These patients have several negative connotations attached to them, which lead to mental health clinicians having a negative attitude toward them; in fact, a minority of clinicians prefer to not work with BPD patients [5].

The pathogenesis of BPD, similar to other psychiatric conditions, is multifactorial, often due to interactions between the environment and genetic components. Moreover, most of the causes are transdiagnostic, which is common among various psychiatric conditions [6]. One promising pathological factor for research is the oxytocinergic system in the brain, the interest in which was triggered by the publication of comparative studies showing low oxytocin levels in BPD patients as compared to controls [7]. This review focuses on the latest literature on the role of oxytocin, its genetics, receptor polymorphism, and how it interacts with the environment and early abuse to cause BPD. This review will also summarize the findings of various clinical trials that used intranasal oxytocin to alleviate BPD symptoms. for the benefit of the general mental health community.

## Review

Method

We searched PubMed for relevant articles on January 25, 2021, by employing the following terms: “Oxytocin” AND “Borderline Personality Disorder”, “Borderline Personality Disorder” AND “Oxytocin”, and “Oxytocin” AND “Borderline*”.

Results

These PubMed searches yielded 62 results. After going through all search results, all review studies were excluded and only original studies were included in this review. After careful consideration and going through all the remaining results, 22 articles were selected, including nine clinical trials, to conduct a literature review to understand the association between oxytocin and BPD.

This review focuses on the latest literature on the role of oxytocin, its genetics, receptor polymorphism, and how it interacts with the environment and early abuse to cause BPD. This review will also summarize the findings from the clinical trials that used intranasal oxytocin to alleviate various BPD symptoms.

Discussion

Oxytocin in Psychoneuroendocrinology

Many neuropeptide hormones have been studied regarding their association with BPD, but the one that has captured our interest is oxytocin. Oxytocin, a neuropeptide hormone, is often portrayed as a love hormone and has been found to have a role in how we perceive ourselves, remember our past relationships, socially interact, and manage stress, especially in the previously mentioned domains, thereby making it an important candidate to be analyzed pertaining to various psychiatric illnesses including BPD [8].

Oxytocin Receptor and Its Genes

Not only are oxytocin levels lower in BPD, but the oxytocin receptor (OXTR) expression is decreased as well, showing the role of oxytocin and its receptor in this disorder [9]. It is important to not see OXTR from an angle of “diathesis-stress”; we should rather see it from an angle of “differential susceptibility” (also known as “plasticity”). Many single nucleotide polymorphisms (SNP) have been identified and linked with BPD, but OXTR rs 53576 having alleles A and G (AA/AG vs. GG) has shown the most consistent results. Differential susceptibility tells us that having a particular allele will make a person more susceptible to positive outcomes in the presence of a positive environment and worse outcomes in the presence of a bad environment. Several studies have identified that people with genotype AA/AG showed a linear relationship of developing BPD with early adverse events, whereas people with genotype GG showed no relationship of developing illness with early adverse events, thus reinforcing the role of the OXTR gene in plasticity [10,11]. It has also been reported that childhood maltreatment in the AA/AG subgroup leads to more empathy towards psychological pain while the GG subgroup remained unaffected by childhood maltreatment; therefore, it is hypothesized that the OXTR gene has a role in disproportionately increased empathy towards psychological pain in BPD patients as compared to controls, with this disproportionality leading to disturbed relatedness. In short, AA/AG genotypes are at lower risk than normal of developing BPD in the presence of early parental warmth, and at the same time, they are at a higher risk than normal of developing psychopathology in presence of early childhood abuse [12].

Role in Neural Circuits

Bottom-up and top-down neural circuitry is often involved in normal emotional processing, and mostly it is the synthesis of these two processes that leads to a balanced approach to emotional reactivity. The top-down pathway includes the prefrontal cortex and its associated regions, whereas the bottom-up pathway is handled by the amygdala and para-amygdala regions. The role of the prefrontal cortex is important in attenuating most of our feelings; moreover, it also helps to make sense of what we feel and why do we feel it in response to some particular stimuli [13]. Patients with BPD show structural and functional abnormalities in a fronto-limbic network with limbic hyperreactivity (more bottom-up pathway), and less control on it from the prefrontal cortex, leading to impulsivity, affective dysregulation, and other core features of BPD [14]. Intranasal oxytocin has been shown to decrease limbic hyperreactivity regardless of scenic valence, with no effects on either the prefrontal cortex or fronto-limbic pathway; hence, it is hypothesized that oxytocin administration can attenuate the interpersonal bias, leading to the aversive interpretation of neutral stimuli and often affective instability. It also reiterates the prosocial and anxiolytic effects of oxytocin on our neural connections [15,16].

Oxytocin and Attachment

The early attachment of a child to a caregiver is an important phase, and caregiver separation in the first five years of the life of a child can lead to disorganized (unresolved) types of attachment later in life, a characteristic pattern of attachment normally seen in the majority of BPD patients [17]. Oxytocin has been shown to have a role in mother-infant bonding as well as social bonding, and hence one can speculate on its association with specific attachment styles [8]. It has been shown in studies that exclusion from an environment can lead to a change in oxytocin levels having a role in alleviating separation pain and mending broken relationships. Borderline patients, specifically the ones with unresolved attachments, have been shown to have a significant decrease in oxytocin levels after separation rather than a normal increase in controls, elucidating the role of oxytocin in higher rejection sensitivity with greater efforts to avoid abandonment in this patient group [18]. This also emphasizes the strong link of oxytocin with respect to the early environment of a child. In a study, borderline patients having a high fear of compassion (FOC) were associated with lower oxytocin levels. High FOC leads to various interpersonal issues among BPD patients. The same set of patients with high FOC had aversive and harsh recalling of their parenting. On the other hand, recalling emotional warmth from parents was positively correlated with higher oxytocin levels in another subset of BPD patients. It again asserts that oxytocin has a greater role in the subset of BPD patients having unresolved attachment issues arising from early childhood abuse [19].

Limitations of Basic Studies

The biggest limitation that could be observed in the studies we reviewed was the methodological inconsistency in finding the correct way to measure oxytocin and the issue as to whether peripheral oxytocin values could relate to central values [20]. The number of patients was fewer in earlier studies, but the latest studies had enough participants to ensure statistical significance [13,15]. It is important to classify BPD patients into phenotypes with prominent core features and dysfunctions since, over time, a certain core feature starts to dominate within a patient, and then study them on the association with oxytocin level and receptor polymorphism to ensure better translational research [3]. It is important to understand that oxytocin has a transdiagnostic role in psychiatric diseases often studied with autism, depression, and personality disorder; therefore, it is important to find out what are the core features that it impacts, and how it forms overall pathology through interaction with other neuropeptides, hormones, and neural connections along with gender differences concerning hormones and neuropeptides [21,22].

Clinical Trials With Oxytocin

To date, there have only been a limited number of studies performed to investigate the therapeutic effects of oxytocin administration on BPD patients (Table 1). These studies have yielded important results regarding the ability of oxytocin to modulate the behavior of patients, although many questions still remain unanswered. In many cases, oxytocin has a prosocial effect on BPD patients, normalizing their defective processing of social cues and thereby improving their interpersonal communication. Significant results to this effect were shown by Bertsch et al. (2013) [23], who showed that a single dose of oxytocin reduced the hyperactivation of the amygdala in BPD patients, thereby normalizing their attention bias to negative social cues. A similar study by Lischke et al. (2017) also demonstrated decreased paralimbic activation in BPD patients following oxytocin administration, although amygdala activity in healthy controls was found to be increased [15]. In a different study, however, Brüne et al. (2013) demonstrated that BPD patients had an avoidant response to angry faces, which was abolished by intranasal oxytocin administration [24]. Increased attention towards threatening stimuli due to the hyperactive amygdala has been linked with increased stress reactivity and cortisol response in BPD patients. Oxytocin was also shown to reduce stress-induced cortisol surge in BPD patients in a study by Simeon et al. (2010) [25]. BPD patients often show issues related to empathy, leading to difficulty in maintaining stable interpersonal relationships. In a study carried out by Domes et al. (2010) [26], oxytocin improved affective empathy and approach motivation in BPD patients. Thus, oxytocin’s ability to improve the processing of social cues, affective empathy, and approach motivation is a promising starting point to investigate its role further in improving dysregulated social behavior in BPD patients. Studies carried out by Schneider et al. (2020) [27] and Brüne et al. (2015) [28] have also demonstrated improvements in dysregulated behavior of BPD patients, by abolishing incongruent and flight behavior respectively, thus making their behavior similar to controls. However, oxytocin does not always have prosocial effects. In two different studies carried out by Bartz et al. (2011) [29] and Ebert et al. (2020) [30], oxytocin was shown to reduce trust among BPD patients. Interestingly, in both these studies, the reduction of trust depended on other features of BPD patients’ personalities. For example, Ebert et al. found a significant correlation between childhood trauma scores and the reduction of trust. Similarly, although oxytocin administration increased trust among anxiously-attached, low-avoidant participants in the study by Bartz et al., it caused a significant decrease in trust among patients with anxiously-attached, rejection-sensitive attachment patterns. These results indicate that oxytocin does not always have a prosocial effect on patients; rather, its effect is modulated by various factors.

There are different reasons as to why oxytocin’s effect on different patients might be different. One possibility is that negative experiences cause dysregulation of the oxytocin system in patients. Oxytocin’s differential effect on different patients may be caused by neurobiological differences, e.g., differences in genetic susceptibility. Another possibility is that oxytocin modulates behavior by increasing the salience of social cues, which may result in triggering a range of different behaviors, both positive and negative, depending on the social context and individual differences in processing different stimuli [31]. Oxytocin’s effect may also depend on the presence of different comorbidities in different patients; thus, oxytocin does not always lead to an improvement in interpersonal behavior in patients with BPD; rather, its effect varies according to a person’s unique characteristics. Although yielding useful results, most of the studies carried out to see the effect of oxytocin on BPD patients are limited in their extent due to their small sample sizes and the fact that most of them consisted of women participants only. Since the effect of exogenous oxytocin may be different in women and men, these studies need to be replicated with larger sample sizes and the inclusion of more men. The effect of comorbidities such as depression, obsessive-compulsive disorder (OCD), eating disorder, and post-traumatic stress disorder (PTSD) on oxytocin’s efficacy also needs to be investigated. 

Many of the BPD patients included in these studies were simultaneously using psychoactive medications [various selective serotonin reuptake inhibitors (SSRIs) or atypical antipsychotics]; therefore, interactions of oxytocin with these medications cannot be excluded. Dose-dependent effects of oxytocin need to be investigated too. These studies did not control for variations in baseline serum oxytocin levels, although these levels tend to vary, especially in people with a history of child abuse. Future studies are warranted to examine more deeply the effect of oxytocin on the behavioral, neurological, and genetic levels, preferably after prolonged and repeated administration or with other adjunct treatments, to more fully explore the potential of oxytocin as a therapeutic agent for BPD patients.

Study limitations

One limitation of this study is that specific keywords related to oxytocin and BPD were searched on only one database (PubMed). Any topics not related to those mentioned above are beyond the scope of this study. In addition, a systematic review of the topic was not completed. Also, a quality assessment of the included studies was not performed. Table 1 presents a summary of studies performed to investigate the therapeutic effects of oxytocin administration on BPD patients.

**Table 1:** A summary of intranasal oxytocin clinical trials M: males; f: female; OT: oxytocin; HC: healthy controls; BPD: borderline personality disorder; SCID-I: Structured Clinical Interview for Diagnostic and Statistical Manual-Fourth Edition Axis I Disorders; SCID-II: Structured Clinical Interview for Diagnostic and Statistical Manual-Fourth Edition Axis II Disorders; IPDE: International Personality Disorder Examination; SSRI: selective serotonin reuptake inhibitor; ECG: electrocardiogram; DSM IV: The Diagnostic and Statistical Manual of Mental Disorders-Fourth Edition

Study	BPD patients: (M + F), average age (years)	Controls: (M + F), average age (years)	BPD assessment	Concurrent medication	Menstrual phase	Exclusion criteria		Time interval between OT administration and analysis of effects	Main findings
						For BPD patients	For controls		
Brüne et al. (2013) [24]	13 (8F + 5M), 28.6 ±7.22	13 (10F + 3M), 25.7 ±6.76	Mini-International Neuropsychiatric Interview	Most patients received psychotropic medication, mainly SSRIs (number unspecified)	All females used oral contraceptives	Excessive smokers; those who participated in another study within 30 days prior to screening; substance dependence; pregnancy; breastfeeding; intending to become pregnant within 30 days of completing the study; prolactin level of >200 ng/ml at baseline; clinically significant ECG abnormalities at screening; acute, serious, or unstable medical conditions	Not specified	45 minutes	Avoidant response to angry faces in BPD patients abolished by OT administration
Domes et al. (2019) [26]	61, age not specified	68, age not specified	Mini-International Neuropsychiatric Interview and SCID-II	27 patients taking antidepressant medication, 9 taking atypical antipsychotics	Mid-luteal phase, free of hormonal contraception for at least 3 months	Pregnant or breastfeeding women; lifetime bipolar disorder; lifetime schizophrenia; current alcohol or substance addiction; patients taking benzodiazepines	Any Axis I and II psychiatric disorder	45 minutes	OT administration normalized affective empathy (particularly for positive stimuli) and approach motivation behavior in BPD patients with differential effect on the groups (more effective in BPD patients)
Schneider et al. (2020) [27]	53F, 30.19 ±7.51	61F, 28.36 ±7.65	SCID-I and IPDE	The participants had to be free of psychotropic medication for at least 2 weeks before participation	No data on hormonal contraception/menstrual phase	Current and lifetime diagnosis of bipolar disorder, schizophrenia, and schizoaffective disorder; alcohol or drug (nicotine excluded) dependence over the last 12 months; pregnancy; severe medical illness; severe visual handicap; neurological disorders; organic brain damage	Not specified	75 minutes	Incongruent behaviors became slower following OT administration in both BPD patients and HC, thus overcoming the deficit in avoidance behavior of BPD patients. No differential effect of OT on different groups
Bertsch et al. (2013) [23]	40F, 24.4 ±4.7	41F, 24.4 ±4.7	SCID-I and II	None	Early follicular phase	IQ less than or equal to 85; pregnancy; endocrinal or neurological disorders; use of any type of regular medication except contraceptives; lifetime diagnoses of schizophrenia, schizoaffective disorder, or bipolar disorder; current alcohol or drug dependence	Current or past psychiatric diagnoses and psychological or psychiatric treatment	45 minutes	OT administration reduced amygdala activation and attention bias to negative social stimuli in BPD patients
Lischke et al. (2017) [15]	51F; placebo: 26.5 ±5.52; oxytocin: 26.43 ±6.24	48F; placebo: 25.25 ±4.41; oxytocin: 23.23 ±2.27	Structured interviews	None	5 HC, 5 BPD patients taking hormonal contraceptives tested during the contraceptive-free period, the rest tested during the early follicular phase of the menstrual cycle	Participants who met DSM-IV diagnostic criteria for schizoaffective disorder, schizophrenia, or intellectual disability; received regular medication within the last 8 weeks or acute medication within the previous week before study enrollment; pregnancy, lactation, menstrual irregularities, or menopause at the time of testing	Participants who met DSM-IV criteria for current or lifetime diagnoses of any Axis I or Axis II disorder; pregnancy, lactation, menstrual irregularities, or menopause at the time of testing	45 minutes	Decreased paralimbic brain activation, normalization of abnormal fixation behavior in BPD patients following OT administration. Increase in insular and amygdala activation following OT administration in HCs, irrespective of emotional stimuli
Simeon et al. (2011) [25]	6F + 8M, 35.1 ±8.0	9F + 4M, 34.5 ±8.9	SCID-I and II	None	Not specified	Lifetime schizophrenia or bipolar I disorder; mental retardation; major medical or neurological illnesses; taking psychotropic or other medications including oral contraceptives within the past 2 weeks (5 weeks for fluoxetine); current major depression, substance use disorder, or eating disorder; currently pregnant, lactating, or menopausal; regular smokers	--	30, 60, and 80 minutes	OT administration abolished the greater rise in dysphoria and cortisol surge in BPD patients following stress with differential effect of oxytocin on stress attenuation
Bartz et al. (2011) [29]	10F + 4M, 35 ±8	6F + 7M, 35 ±8	SCID-I and II	None	Not specified	Psychotropic or other medications for at least 2 weeks prior to the study (5 weeks for fluoxetine); current substance use disorder, major depression or eating disorders (anorexia or bulimia); lifetime schizophrenia or bipolar I disorder; mental retardation and medical or neurological illness; pregnant, lactating, or menopausal females	Any lifetime Axis I or II disorders	35 minutes	OT resulted in significantly less trusting expectations and cooperation for anxiously-attached, rejection-sensitive participants, but no effect on less anxiously-attached participants, OXT promoted cooperation in anxiously-attached, low-avoidant participants (intimacy seekers)
Ebert et al. (2013) [30]	5M + 8F, 28.6	3M + 10F, 25.7	SCID	11 patients taking SSRI/melatonergic antidepressants	All female participants used oral contraceptives	Pregnancy; breastfeeding; addiction to alcohol or illegal drugs; excessive smoking; ECG abnormalities; recent illnesses; stable psychopharmacological medication was accepted if remained unchanged between sessions	Any psychiatric disorders	30 minutes	Decreased trust in highly traumatized patients following OT administration
Brüne et al. (2015) [28]	5M + 10F, 27.5 ±7.3	5M + 10F, 25.7 ±6.4	SCID-I and II	11 patients taking SSRI/melatonergic antidepressants	Not specified	Excessive smoking; participation in another study within 30 days before screening; a history of substance dependence; pregnancy or intention to become pregnant within 30 days of completing the study; current breastfeeding; prolactin level of >200 ng/ml at baseline; clinically significant ECG abnormalities; any acute, serious, or unstable medical condition	Any psychiatric disorders	Not specified	OT increased affiliative behavior in controls when given at T1 but not T2. No such effect was seen in BPD patients. Flight behavior was diminished in both HC and BPD after OT administration at T1 but not at T2

## Conclusions

In this study, we tried to capture the role of oxytocin in the pathogenesis of BPD through a review of the relevant clinical trials. Like all psychiatric diseases, an eclectic model towards BPD has to be considered, which will include genetics, gene-environment interactions, and psychological and sociological factors. Oxytocin is an important factor in all of these domains. Our study also delineates the importance of gene-environment interactions and the role of the oxytocinergic system in plasticity (susceptibility model). Based on the various studies we examined, no one hit model can be devised for BPD; rather, various factors combine together to finally lead to an individual towards this disorder. Further studies of these factors along with oxytocin and other neuropeptide hormones can help us to understand the pathogenesis and develop new therapeutic models to treat the morbidity of BPD. We have pointed out that specific phenotypes of BPD having unresolved attachment have greater defects in the oxytocinergic system as compared to other BPD phenotypes. Similarly, clinical trials have shown that some symptoms improved more than others in various BPD patients. These findings provide us with a good avenue to perform more studies on personalized medicine to target specific domains responsive to oxytocin adjunct with other psychoactive medications and psychotherapies.
